# Supplementary material for: Wildfire, ecosystem, and climate interactions in the Early Triassic
Source: Commun Earth Environ. 2025 Oct 21;6(1):830. doi: 10.1038/s43247-025-02789-x (PMC12540185; doi:10.1038/s43247-025-02789-x)
Supplement: Supplementary file 1 — Supplementary Information [file 43247_2025_2789_MOESM1_ESM.pdf]

Supplementary Material

Wildfire, ecosystem and climate interactions in the Early Triassic

Franziska R. Blattmann[a,b*], Charline Ragon[c], Torsten W. Vennemann[a], Elke Schneebeli-Hermann[d], Christian Vérard[e], Jérôme Kasparian[c], Maura Brunetti[c], Hugo F.R. Bucher[d], Thierry Adatte[f], and Clayton R. Magill[g]

[a] Institute of Earth Surface Dynamics, University of Lausanne, Quartier UNIL-Mouline, 1015 Lausanne, Switzerland

[b] Department of Geoscience, Aarhus University, Høegh-Gulbergs Gade 2, Aarhus C, Denmark

[c] Group of Applied Physics and Institute for Environmental Sciences, University of Geneva 1205 Geneva, Switzerland

[d] Department of Paleontology, University of Zürich, Karl-Schmid-Strasse 4, 8006 Zürich, Switzerland

[e] Section of Earth and Environmental Sciences, University of Geneva, 1205 Geneva, Switzerland

[f] Institute of Earth Sciences, University of Lausanne, Quartier UNIL-Mouline, 1015 Lausanne, Switzerland

[g] The Lyell Centre, Heriot-Watt University, Edinburgh EH14 4BA United Kingdom

[*]Corresponding author

Franziska R. Blattmann, *Franziska.Blattmann@geo.au.dk*

This supplemental material file contains five supplemental figures and three supplemental table that gives supporting evidence and analytical context to Blattmann et al. (2025) manuscript titled Wildfire, ecosystem and climate interactions in the Early Triassic.

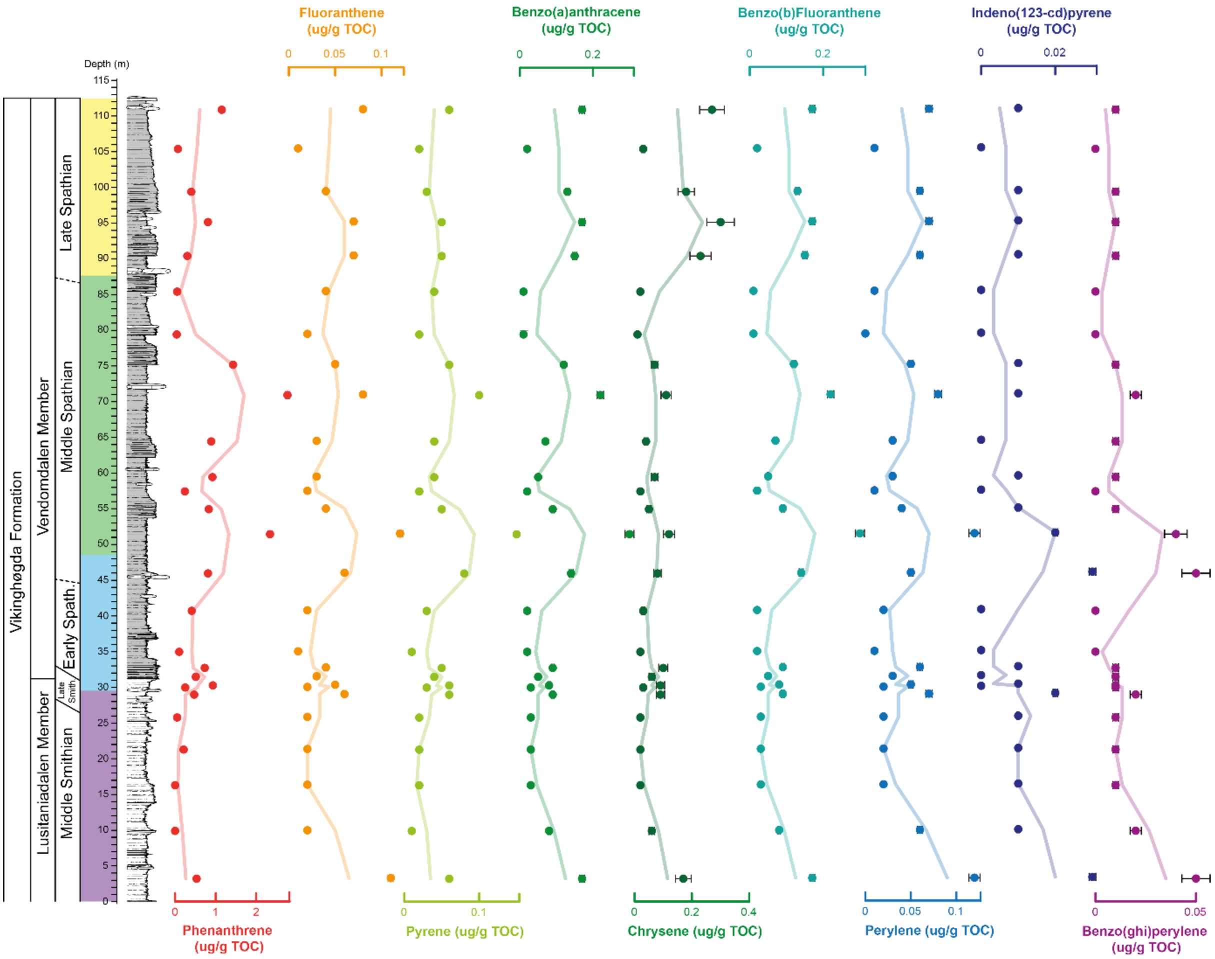

Figure S1. Quantification of all measured non-alkylated PAHs versus depth. The light shaded line represents a 3-point moving average.

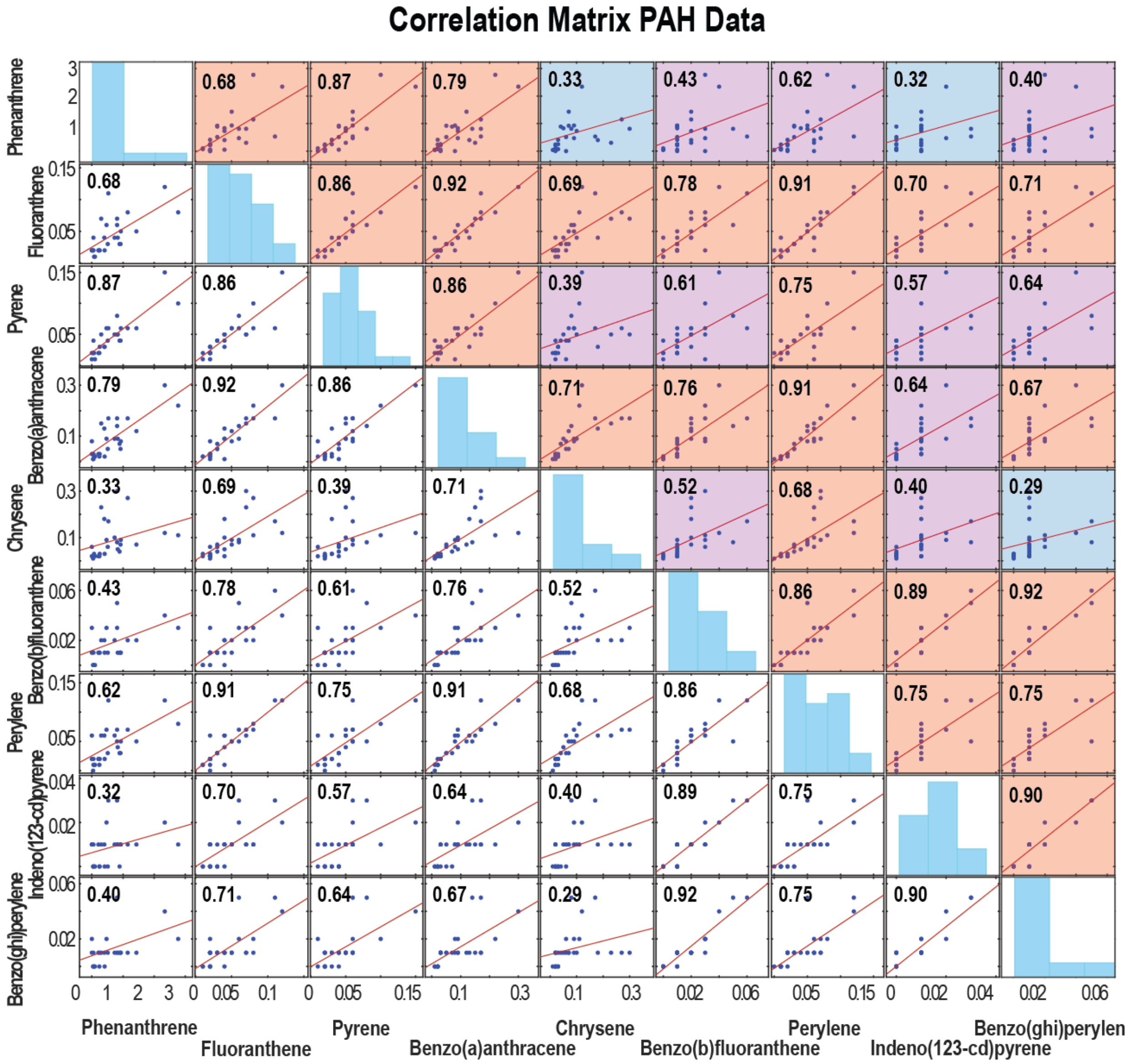

Figure S2. Correlation matrix of all non-alkylated PAHs with red indicating high correlation, purple medium correlation and blue indicating low correlation.

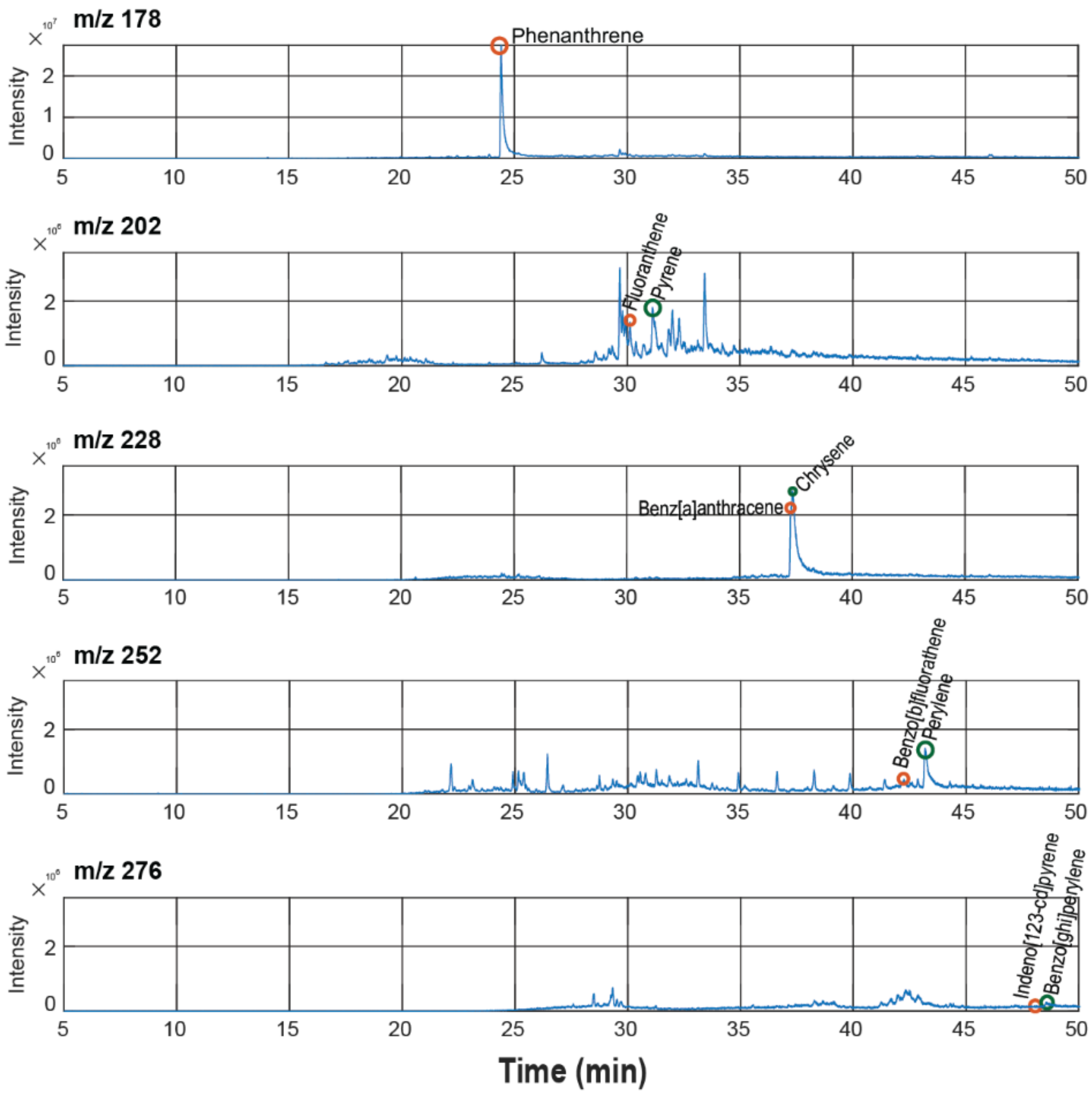

Figure S3. Chromatogram of all non-alkylated PAHs, sample STA BM 8, In m/z 178 phenanthrene was identified while anthracene cannot be exclusively distinguished. In m/z 202 fluoranthene and pyrene were identified; in m/z 228 benz[a]anthracene and chrysene were identified; in m/z 252 benzo[b]fluoranthene and perylene were identified; in m/z 276 indeno[123-cd]pyrene and benzo[ghi]perylene. All PAH identification was confirmed with the NIST mass spectral 2016 database. See Karp, et al. [1], Fox, et al. [2], Grice, et al. [3], Nabbefeld, et al. [4] for more information on compound identification.

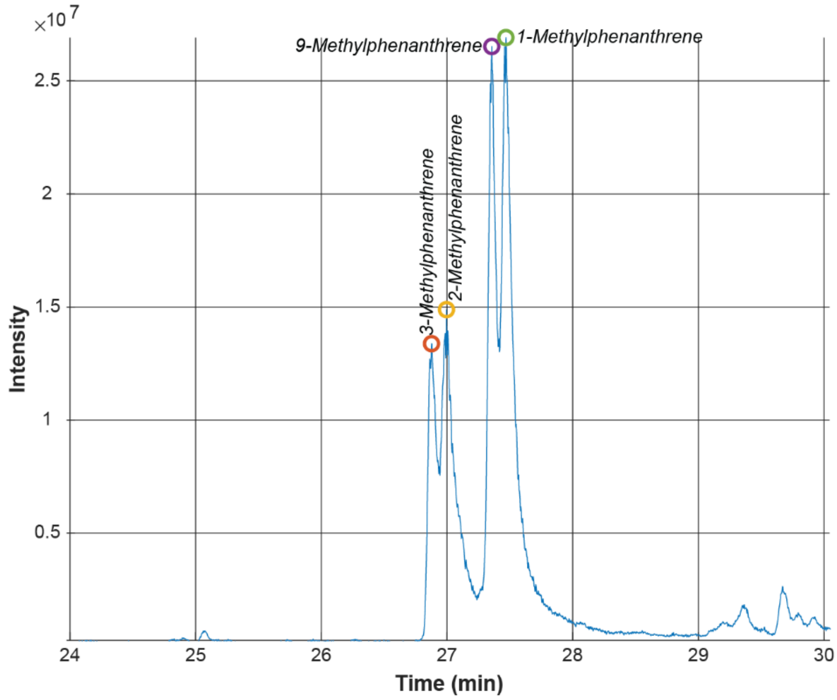

Figure S4. Chromatogram of methylphenanthranes, SIM Channel *m/z* 192, samples STA BM 8. See Radke and Welte [5] for more information on identification of methylphenanthranes.

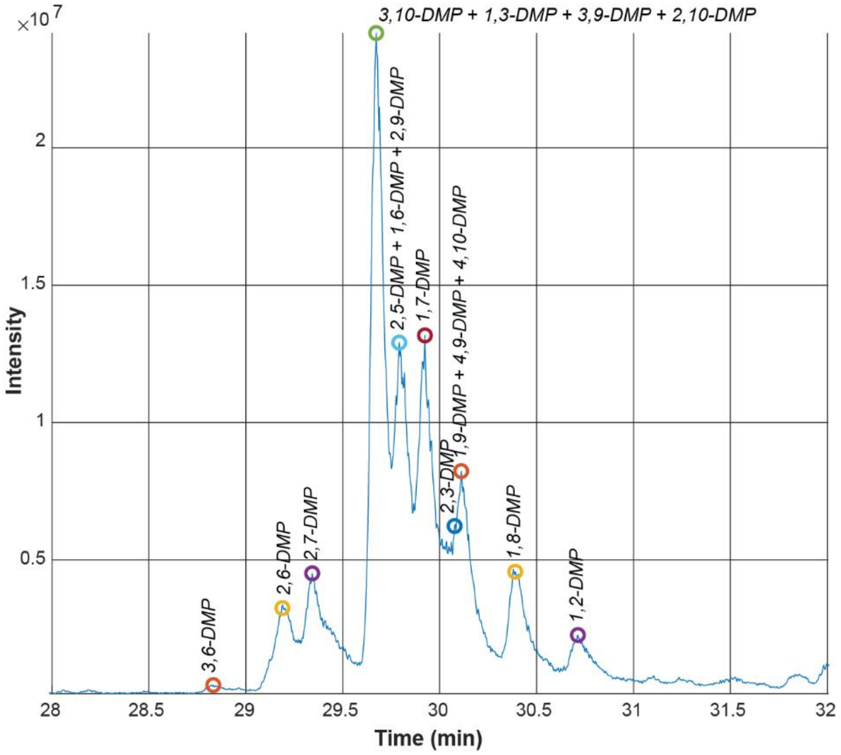

Figure S5. Chromatogram of dimethylphenanthranes (DMP), SIM Channel *m/z* 206, sample STA BM 8. See Karp, et al. [1] and Kappenberg, et al. [6] for more information on identification of DMP.

Table S1. PAH and n-alkane ratio calculations.

Proxy or Ratio	Calculation	Diagnostic Value	Reference
MPI-1	$MPI\text{-}1 = \frac{1.5 * (2MPh + 3MPh)}{(PHE + 1MPh + 9MPh)}$	See Figure 3A	Radke and Welte [5]
Terrestrial-Aquatic Ratio (TAR)	$TAR = \frac{(C_{25} + C_{27} + C_{29} + C_{31})}{(C_{15} + C_{17} + C_{19})}$	Terrigenous: TAR > 4<br>Aquatic: TAR < 1	Bourbonniere and Meyers [7]
LMW/Total PAHs	$\frac{LMW}{Total} = \frac{PHE + FLA + PYR}{Total\ PAHs}$	Residue: 0.35-0.8<br>Smoke: 0.75-0.95	Karp, et al. [1]

Table S2. RockEval data containing TOC (wt.%), Hydrogen Index (HI), Oxygen Index (OI) and Tmax (°C).

Name	TOC [wt.%]	HI [mg HC/g TOC]	OI [mg CO2/g TOC]	Tmax [°C]
STA BM 1	0.61	171	42	438
STA BM 2	1.04	204	49	439
STA BM 3	0.8	143	62	439
STA BM 4	0.63	174	65	439
STA BM 5	1.02	161	57	439
STA BM 6	0.63	169	91	437
STA BM 7	1.32	312	31	437
STA BM 8	2.46	401	14	439
STA BM 9	2.75	421	22	439
STA BM 10	1.44	294	23	438
STA BM 11	3.01	228	28	437
STA BM 12	2.19	287	23	438
STA BM 13	1.59	264	22	437
STA BM 14	1.44	263	23	437
STA BM 15	1.83	270	19	438
STA BM 16	1.59	267	24	437
STA BM 17	1.92	255	23	438
STA BM 18	3.24	322	13	439
STA BM 19	1.52	278	23	438
STA BM 20	2.13	297	15	439
STA BM 21	1.93	288	17	436
STA BM 22	2.16	269	23	439
STA BM 23	3.85	367	18	440
STA BM 24	2.29	273	23	438
STA BM 25	2.41	337	20	439
STA BM 26	4.31	408	9	438
STA BM 27	3.77	383	15	437

Table S3. Overview of analyzed PAH biomarkers.

**Name**	**Abbreviation**	**Molecular Structures**	**SIM Channel**
Phenanthrene	PHE	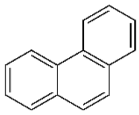	m/z 178
Methylphenanthrenes	MPh		m/z 192
Dimethylphenanthrane	DMP		m/z 206
Fluoranthene	FLA	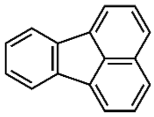	m/z 202
Pyrene	PYR	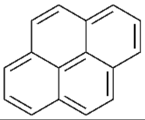	m/z 202
Benz[a]anthracene	BaA	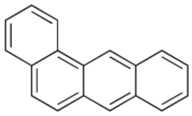	m/z 228
Chrysene	CHR	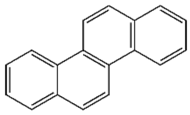	m/z 228
Benzo[b]fluoranthene	BbF	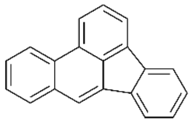	m/z 252
Perylene	PER	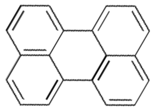	m/z 252
Indeno[123-cd]pyrene	IcdP	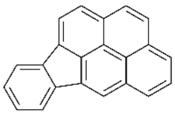	m/z 276
Benzo[ghi]perylene	BghiP	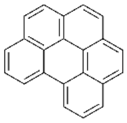	m/z 276

Supplementary References

1 Karp, A. T., Holman, A. I., Hopper, P., Grice, K. & Freeman, K. H. Fire distinguishers: Refined interpretations of polycyclic aromatic hydrocarbons for paleo-applications. *Geochimica et Cosmochimica Acta* **289**, 93-113 (2020). https://doi.org/10.1016/j.gca.2020.08.024

2 Fox, C., Whiteside, J., Olsen, P. & Grice, K. Flame out! End-Triassic mass extinction polycyclic aromatic hydrocarbons reflect more than just fire. *Earth and Planetary Science Letters* **584**, 117418 (2022). https://doi.org/10.1016/j.epsl.2022.117418.

3 Grice, K., Nabbefeld, B. & Maslen, E. Source and significance of selected polycyclic aromatic hydrocarbons in sediments (Hovea-3 well, Perth Basin, Western Australia) spanning the Permian–Triassic boundary. *Organic Geochemistry* **38**, 1795-1803 (2007). https://doi.org/10.1016/j.orggeochem.2007.07.001

4 Nabbefeld, B., Grice, K., Summons, R. E., Hays, L. E. & Cao, C. Significance of polycyclic aromatic hydrocarbons (PAHs) in Permian/Triassic boundary sections. *Applied Geochemistry* **25**, 1374-1382 (2010). https://doi.org/10.1016/j.apgeochem.2010.06.008

5 Radke, M. & Welte, D. in *Advances Organic Geochemistry 1981 : proceedings of the 10th International Meeting on Organic Geochemistry, University of Bergen, Norway, 14-18 September 1981* (ed Malvin Bjorøy) 504-512 (Wiley, 1983).

6 Kappenberg, A., Braun, M., Amelung, W. & Lehndorff, E. Fire condensates and charcoals: Chemical composition and fuel source identification. *Organic Geochemistry* **130**, 43-50 (2019). https://doi.org/10.1016/j.orggeochem.2019.01.009

7 Bourbonniere, R. A. & Meyers, P. A. Sedimentary geolipid records of historical changes in the watersheds and productivities of Lakes Ontario and Erie. *Limnology and Oceanography* **41**, 352-359 (1996). https://doi.org/10.4319/lo.1996.41.2.0352
